# Overall survival of patients with recurrent pancreatic cancer treated with systemic therapy: a retrospective study

**DOI:** 10.1186/s12885-019-5630-4

**Published:** 2019-05-17

**Authors:** Olumide B. Gbolahan, Yan Tong, Amikar Sehdev, Bert O’Neil, Safi Shahda

**Affiliations:** 1 Department of Hematology Oncology, Indiana University School of Medicine, 535 Barnhill Drive, RT 473, Indianapolis, IN 46202 USA; 2 Department of Biostatistics-HS3000, Indiana University School of Medicine, 410 West 10th; Street, Indianapolis, IN 46202 USA; 3 Department of Hematology Oncology, Indiana University School of Medicine, Indiana Cancer; Pavilion, 535 Barnhill Drive, Indianapolis, IN 46202-5289 USA; 4 Indiana University School of Medicine, Indiana Cancer Pavilion; 535 Barnhill Drive, Indianapolis, IN 46202-5289 USA; 5 Department of Hematology Oncology, Indiana University School of Medicine, Indiana Cancer Pavilion, Suite 130-C 535; 535 Barnhill Drive, Indianapolis, IN 46202-5289 USA

**Keywords:** Recurrent pancreatic ductal adenocarcinoma, Recurrent pancreatic cancer, Metastatic pancreatic cancer, FOLFIRINOX, Gemcitabine-nab paclitaxel, Combination chemotherapy

## Abstract

**Background:**

Only a few patients with pancreatic ductal adenocarcinoma (PDAC) recurring after curative resection and peri-operative (neoadjuvant and adjuvant) therapy are included in clinical trials of metastatic PDAC. As such, there is a paucity of data to guide treatment after relapse, and patients are treated similarly to those with de novo metastatic PDAC (mPDAC). We evaluated the patterns of chemotherapy use and over-all survival (OS) in patients with recurrent PDAC (rPDAC) following curative therapy.

**Methods:**

In this retrospective study, the Indiana University pancreatic cancer database was used to identify patients with PDAC who underwent curative resection and subsequently developed recurrence. Demographics, tumor and treatment characteristics were collected. Patients were broadly divided into those who received chemotherapy for rPDAC and those who did not. Patients in the former category were further subdivided into those who received single agent therapy, any standard combination therapy (5-fluorouracil/irinotecan/oxaliplatin combination or gemcitabine/nab-paclitaxel) and those who received non-standard combinations. Survival analysis was performed by the Kaplan-Meier method. Log rank tests were used to determine differences in survival between treated rPDAC patients and those not treated. Cox regression analysis was employed to evaluate factors associated with OS.

**Results:**

We identified 435 patients with resected PDAC treated between 2008 and 2014. Two hundred and twenty-three patients (51.2%) were diagnosed with rPDAC. Of these, 140 patients (63%) received chemotherapy whereas 71 patients (32%) did not receive chemotherapy. The 74 patients (53%) who received any standard, approved multiagent combination regimen had a median OS of 14 months compared to 8 months for the 47 patents (34%) who received other non-standard combinations and the 19 (13%) who received single agent therapy (*P* = 0.029). Multivariate cox regression analysis showed that margin negative resection, peri-operative therapy, radiotherapy and the use of any chemotherapy for rPDAC were associated with improved OS.

**Conclusion:**

Our findings support the use of standard approved multi-agent therapy in rPDAC. Patients derive significant benefit from these standard combination therapies with median OS that is comparable to what is observed with treatment for de novo mPDAC.

**Electronic supplementary material:**

The online version of this article (10.1186/s12885-019-5630-4) contains supplementary material, which is available to authorized users.

## Background

The OS of patients with PDAC has slowly improved from the often quoted 5 to 8.3% recently [1] . Nevertheless, PDAC remains the 4th leading cause of cancer related death in the United States and is projected to become the second leading cause within a decade [2, 3]. Among the 10–20% of patients who present with resectable disease, adjuvant therapy with fluorouracil or gemcitabine doubles the surgical cure rate [4, 5] and more recently, the addition of capecitabine to gemcitabine led to a 5-year survival of nearly 30% [6]. In spite of these improvements, up to 80% of patients with rPDAC who receive curative intent therapy will relapse with local and/or distant disease, which would be associated with mortality within 2 years from diagnosis [7, 8].

In the pivotal randomized phase III studies that established combination regimens for metastatic PDAC, only a few patents had rPDAC [9–11]. For instance, in the MPACT study of gemcitabine and nab-paclitaxel (GnP), only 7% of enrollees had previously received a Whipple procedure and 4% received adjuvant therapy. In the ACCORD trial, the authors did not specify that percentage although 5 and 8% in the gemcitabine and FOLFIRINOX arms respectively had metachronous metastasis. While it is standard at this time to treat rPDAC similarly to de novo mPDAC, there are few data regarding outcomes using combination chemotherapy in rPDAC. The concerns about the tolerability of these regimens have been documented [12] and these may be amplified in patients who have recently undergone a pancreaticoduodenectomy [13]. Furthermore, it is not clear whether these patients obtain similar benefit from chemotherapy as patients with de novo mPDAC.

The objective of this study was to describe the outcome of patients with rPDAC who received single-agent and combination chemotherapy regimens after recurrence.

## Methods

### Study design and population

We conducted a retrospective study using Indiana University’s Institution Review Board (IRB) approved database of patients with PDAC [14]. Information in this database are gathered retrospectively on an ongoing basis and stored in the OnCore™ Enterprise Research System, in compliance with institutional guidelines. Data are collected from patients’ electronic medical records (EMR) including clinic visits, radiology and pathology reports. The current study was approved by the Indiana University’s IRB. All patients included in the study had resected PDAC and subsequently developed recurrent disease (local, distant or both). Patients with rPDAC without adequate follow-up information or treatment data after recurrence were excluded from the final analysis.

### Data collection

Data on patients with resected PDAC who received any care (medical, surgical and/ or second opinion evaluation) between 2008 and 2014 at the Indiana University Simon Cancer Center gastrointestinal oncology clinics were retrieved from the OnCore™ data repository. Demographic information, tumor and treatment characteristics were collected and confirmed by an independent review of the patients’ EMR. The data collected included; age at diagnosis, gender, ethnicity, and co-morbid conditions (including diabetes and other cardiovascular disease). Perioperative therapy and treatments offered following recurrence were also recorded. Tumor characteristics such as anatomic location, tumor stage, resection margin status and the presence of lymphovascular and perineural invasion, were collected. PDAC recurrence was defined by the treating physician based on a combination of clinical symptoms, rising CA 19–9 titers and/or radiographic progression (whether local, distant or a combination of both) while patients received standard of care routine follow-up.

The type of chemotherapy received for rPDAC was broadly categorized into single agent therapy, standard combination with either gemcitabine and nab-paclitaxel (GnP) or FOLFIRINOX and other non-standard combinations (such as FOLFOX). Because patients may have received multiple lines of therapy, we defined a line of therapy as administration of a single active agent or combination therapy as described above. The addition of an active agent to a prior single agent or combination regimen or a switch to a different single agent/combination constituted a change in line of therapy while discontinuation of an agent from a combination did not qualify as a change in therapy.

This definition was adopted prior to data analysis and is an adaptation of the definition of the lines of therapy reported in the metastatic colorectal cancer literature [15]. Patients who received both a standard and non-standard combination among their lines of therapy were categorized for analysis as receiving standard therapy. Data used for analysis were reviewed and are current till the 31st of March 2017.

### Study objectives

The primary objective of this study was to estimate the OS for patients with rPDAC who received chemotherapy. We determined OS for those who received single agent, standard and non-standard combination regimens. The OS was defined as the time from institution of chemotherapy following disease recurrence to the date of death or last follow up. All subsequent references to OS describe a post recurrence survival period unless otherwise stated. We also sought to determine the patterns of use of combination chemotherapy for rPDAC patients.

### Statistical analysis

Demographic and clinical characteristics were summarized and compared between patients who received and did not receive chemotherapy for rPDAC. Log-rank tests were used to examine the difference in OS between the different chemotherapy groups, and Kaplan-Meier estimates of OS were plotted. Univariate Cox proportional hazard regression models were used to determine factors associated with OS. Variables significant at a *P*-value < 0.25 in the univariate analysis were included in multivariate analysis. All data were truncated at the date of death, date of last follow up or the 31st of March 2017, the cutoff date for the analysis.

Data were collected from the OnCore™ Enterprise system to a Microsoft Excel spread sheet and analyzed with the SAS statistics software application v 9.4 (SAS Institute Inc., Cary, NC).

## Results

### Patients characteristics

**Fig. 1:**
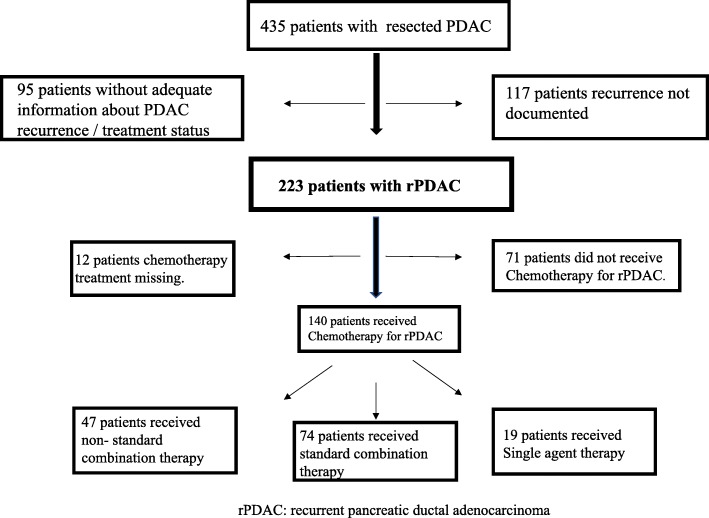
Flow chart showing the study population. Four hundred and thirty-five patients were identified from the database of patients with localized and resected pancreatic cancer. Of the 223 with recurrent pancreatic ductal adenocarcinoma, 140 received systemic hemotherapy and 71 did not

Four hundred thirty-five patients with resected PDAC were reviewed for this study (Fig. 1). Of these, 223 (51%) had documented recurrence (rPDAC) and sufficient follow-up information for final analysis by the data cut-off date. Of the 223 patients, 140 (63%) patients received systemic chemotherapy, and these constituted the final cohort of this study. Fifty-six percent of these were male, and the median age at diagnosis was 64.3 years. Thirteen patients (6%) had stage I disease, 13% had stage IIA PDAC and 80% had Stage IIB disease at the time of diagnosis (Table 1). The median serum Ca 19–9 at the time of recurrence was 332.5 U/ml (range (0–140,000 U/ml). Sixty patients (27%) had local recurrence only and 162 (73%) had distant recurrence. The median time from diagnosis to recurrence was 11 months (95% CI 10–11 months).

**Table 1:** Baseline characteristics of patients with recurrent pancreatic ductal adenocarcinoma

Characteristic	*Overall *N* = 211	No Therapy *N* = 71	Non-Standard Combination *N* = 47	Single Agent *N* = 19	Standard Combination *N* = 74	*P*-value
Age at diagnosis (years) Median (range)	64.3 (57.2, 70.6)	67.9 (36.4, 87.2)	64.2 (45.7, 82.7)	64.6 (44.2, 88.9)	61.7 (31.6, 83.9)	.0079^1^
Gender						.0675
F	96 (45%)	38 (54%)	19 (40%)	12 (63%)	27 (36%)	
M	115 (55%)	33 (46%)	28 (60%)	7 (37%)	47 (64%)	
Race						.7211
Asian	1 (0%)	0	0	0	1 (1%)	
Black or African American	9 (4%)	3 (4%)	1 (2%)	2 (11%)	3 (4%)	
Other	1 (0%)	1 (1%)	0	0	0	
White	200 (95%)	67 (94%)	46 (98%)	17 (89%)	70 (95%)	
Stage						.0293
I	12 (6%)	2 (3%)	4 (9%)	2 (11%)	4 (6%)	
IIA	27 (13%)	6 (8%)	6 (13%)	3 (16%)	12 (17%)	
IIB	166 (80%)	63 (89%)	36 (78%)	12 (63%)	55 (76%)	
III	3 (1%)	0	0	2 (11%)	1 (1%)	
Comorbid Conditions						.6393
No	133 (63%)	43 (61%)	32 (68%)	10 (53%)	48 (65%)	
Yes	78 (37%)	28 (39%)	15 (32%)	9 (47%)	26 (35%)	
Diabetes						.3180
No	131 (62%)	41 (58%)	28 (60%)	10 (53%)	52 (70%)	
Yes	80 (38%)	30 (42%)	19 (40%)	9 (47%)	22 (30%)	
Tumor Location						.7085
Body	10 (5%)	3 (4%)	2 (4%)	2 (11%)	3 (4%)	
Head	153 (73%)	54 (76%)	37 (79%)	14 (74%)	48 (65%)	
Neck	14 (7%)	4 (6%)	1 (2%)	1 (5%)	8 (11%)	
Tail	25 (12%)	8 (11%)	4 (9%)	2 (11%)	11 (15%)	
Uncinate Process	9 (4%)	2 (3%)	3 (6%)		4 (5%)	
Margin Negative Resection						.8253
No	51 (24%)	18 (26%)	12 (26%)	3 (16%)	18 (24%)	
Yes	158 (76%)	52 (74%)	34 (74%)	16 (84%)	56 (76%)	
Lymphovascular Invasion						.1892
No	67 (33%)	18 (26%)	13 (30%)	5 (26%)	31 (42%)	
Yes	139 (67%)	51 (74%)	31 (70%)	14 (74%)	43 (58%)	
Perineural Invasion						.9466
No	29 (14%)	9 (13%)	7 (15%)	2 (11%)	11 (15%)	
Yes	180 (86%)	61 (87%)	39 (85%)	17 (89%)	63 (85%)	
Recurrence Site						.8993
Distant	154 (73%)	51 (72%)	36 (77%)	13 (68%)	54 (74%)	
Local	56 (27%)	20 (28%)	11 (23%)	6 (32%)	19 (26%)	
Perioperative Chemotherapy						.0088
No	41 (19%)	21 (30%)	2 (4%)	4 (21%)	14 (19%)	
Yes	170 (81%)	50 (70%)	45 (96%)	15 (79%)	60 (81%)	
Radiotherapy for rPDAC						.8576
No	183 (89%)	61 (87%)	41 (89%)	15 (94%)	66 (90%)	
Yes	22 (11%)	9 (13%)	5 (11%)	1 (6%)	7 (10%)	

rPDAC: recurrent pancreatic ductal adenocarcinoma. N: number

*Perioperative chemotherapy: combination of patients who received neoadjuvant or adjuvant chemotherapy

^1^*P*-value calculated with the ANOVA test

In addition, 61 patients (27%) with rPDAC recurred within 6 months of surgery for primary disease, which covers the usual period during which adjuvant therapy is delivered. Forty-one of these patients with early recurrence had margin negative resection but 49 had tumor samples with lymphovascular invasion.

### Treatments

Following recurrence, 140 of the 223 patients (63%) received systemic. Nineteen (13%) received single agent therapy only, 74 (53%) received standard combination treatment and 47 (34%) received only non-standard combinations. Seventy-three (52%) patients received only one line of therapy and 67 (48%) received 2 or more lines of therapy (Table 2). Based on univariate logistic regression (with a *P* value set at < 0.25); female gender, lymphovascular invasion, presence of local recurrence and perioperative chemotherapy were associated with a lack of administration of systemic therapy at the time of recurrence. Following multivariate modeling of these variables, only female gender remained significantly associated (OR 1.95, 95% CI, 1.02–3.70, *P* = 0.00418) with a lack of chemotherapy administration for rPDAC (Additional file 1: Table S1 and Table S2).

**Table 2:** Treatment administered to patients with recurrent pancreatic ductal adenocarcinoma

Characteristics	Number = 140 (%)
Chemotherapy for rPDAC	
Single Agent	19 (13)
Non-Standard Combination	47 (34)
Standard Combination	74 (53)
Lines of chemotherapy for rPDAC	
1	73 (52)
2	47 (34)
3	15 (11)
4–6	5 (3)

rPDAC: recurrent pancreatic ductal adenocarcinoma

Radiation therapy was infrequently used in this cohort. Although 60 patients with rPDAC had local recurrence, only 11 patients (18%) received radiation therapy, mean radiation 5080 Gy. Another 11 (7%) with distant metastatic recurrence received radiation (mean 3050 Gy). Focusing on the 140 patients who received systemic therapy, local recurrence was reported in 36 patients, only 7 of these received radiation, median dose 5040Gy (range 5000-5040Gy). Six patients with distant metastasis received radiation (median 3375Gy, range 2400 – 6000Gy).

### Survival outcomes and associated factors

**Fig. 2:**
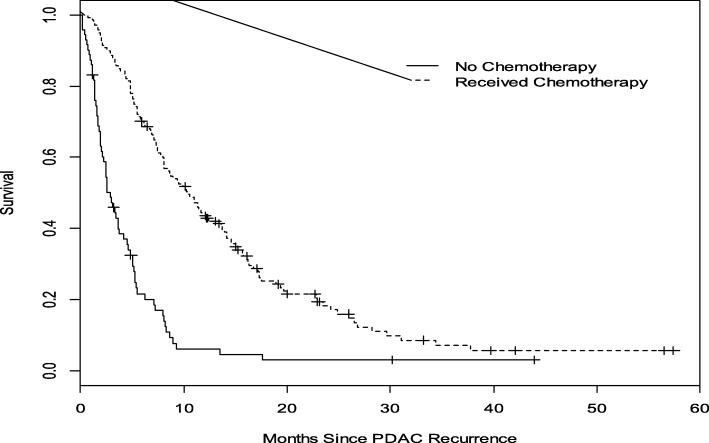
Overall Survival analysis of rPDAC patients who received chemotherapy compared to those who did not. The median OS for those who received chemotherapy (broken lines) was 10 months compared to 3 months without chemotherapy (solid line). Log—rank *p* < 0.0001

**Fig. 3:**
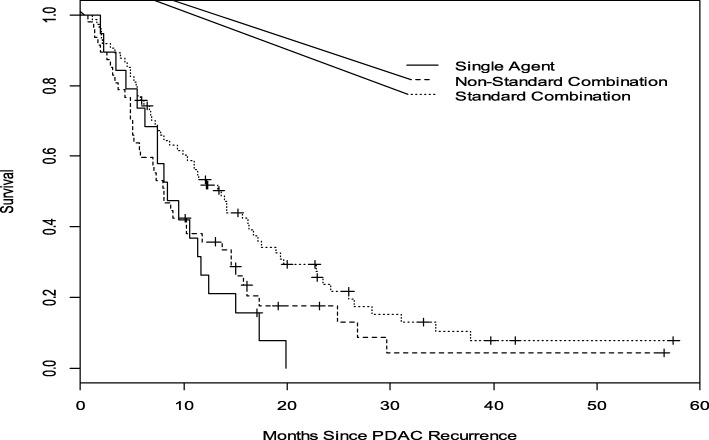
Overall Survival Analysis of rPDAC patients who received different chemotherapy regimens. Patients who received standard combination; FOLFIRINOX and /or gemcitabine- nab-paclitaxel (tiny broken lines) compared to those who received other non-standard combinations (larger broken lines) and single agent therapy (solid line). The median OS; standard combinations was 14 months, non-standard and single agent therapy, 8 months. Log-rank *p* = 0.0295

**Fig. 4:**
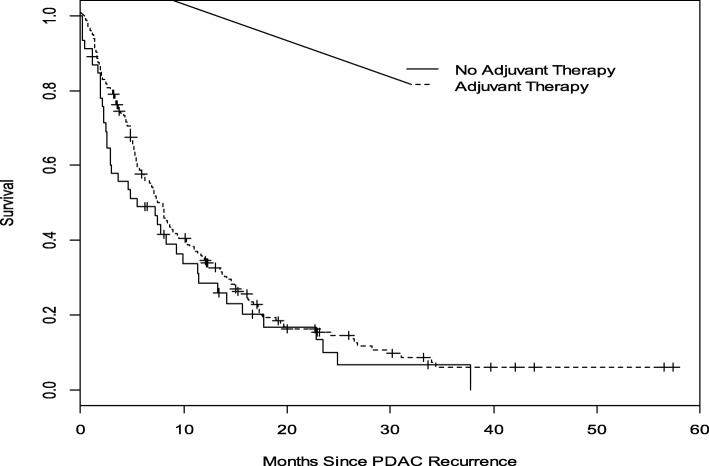
Overall survival analysis of rPDAC patients who received *peri-operative chemotherapy compared to no peri-operative therapy. The median OS for patients who received peri-operative chemotherapy (broken line) was 8 months and 5 months for those who did not receive perioperative chemotherapy (solid line). Log—rank *p* < 0.2206. ^*^perioperative (both adjuvant and neoadjuvant therapy)

The median post-recurrence OS (mOS) for all patients with rPDAC was 7 months (95% CI, 6–9 months). Compared with a mOS of 3 months (95% CI 2–4 months) for patients who did not receive any chemotherapy, patients who received chemotherapy had a mOS of 10 months (95% CI 8–13 months, *P* < 0.0001) (Fig. 2). Administration of standard combination therapy regimens was associated with a significant improvement in OS compared to single agent and non-standard combination chemotherapies (median OS 14 months [95% CI 9–17] versus 8 months [95% CI 6–12 months] versus 8 months [95% CI 5–12 months], *P* = 0.029; Fig. 3). Median OS did not significantly differ between the cohort that received perioperative therapy (8 months, 95% CI 6–9 months) and those who did not (5 months, 95% CI 3–10 months), *P* = 0.2206. (Fig. 4). Furthermore, of the 140 patients who received chemotherapy, there was no difference in OS between those with distant recurrence (*N* = 103) and the 36 with local recurrence; mOS 10 months (95% CI, 7–12 months) vs 12 months (95% CI, 8–17 months) *P* = 0.4092.

Table 3 lists factors associated with improved overall survival in univariate analysis, and multivariate Cox regression of these variables (Table 4) showed that margin negative resection, perioperative therapy, radiation therapy and chemotherapy for rPDAC were associated with improved post recurrence survival.

**Table 3:** Univariate analysis of factors associated with overall survival

Factor	Comparison	Hazard Ratio	Lower 95%CL	Upper 95%CL	*P*-value
Gender	F vs. M	1.02	0.77	1.36	0.8857
Pathologic Stage	IIA vs. I	1.08	0.51	2.32	0.8368
	IIB vs. I	1.77	0.93	3.37	0.0805
Chemotherapy for rPDAC	Yes vs. No	0.31	0.23	0.43	<.0001
Margin Negative	Yes vs. No	0.75	0.54	1.04	0.0860
Lymphovascular Invasion	Yes vs. No	1.53	1.11	2.10	0.0089
Perineural Invasion	Yes vs. No	0.97	0.64	1.46	0.8731
Co-morbid Conditions	Yes vs. No	1.11	0.83	1.50	0.4805
Diabetes Mellitus	Yes vs. No	0.90	0.67	1.22	0.5040
Radiotherapy for rPDAC	Yes vs. No	0.57	0.35	0.92	0.0207
Recurrence site	Distant vs. Local	1.15	0.83	1.59	0.3979
^a^Perioperative therapy	Yes vs. No	0.80	0.56	1.14	0.2220
rPDAC chemotherapy group	Non-Standard Combination vs. Single Agent	0.82	0.47	1.43	0.4764
rPDAC chemotherapy group	Standard Combination vs. Single Agent	0.54	0.31	0.92	0.0246
Ca19–9 at recurrence	<1000 U/ml vs >1000 U/ml	1.007	0.999	1.016	0.0914
CA19–9 at diagnosis	<1000 U/ml vs >1000 U/ml	1.073	1.038	1.108	<.0001

*rPDAC* recurrent pancreatic ductal adenocarcinoma

^a^Perioperative therapy: combination of patients who received neoadjuvant or adjuvant therapy

**Table 4:** Multivariate Cox Regression Model of factors associated with overall survival^a^

Factor	Comparison	Hazard Ratio	Lower 95%CL	Upper 95%CL	*P*-value
Pathologic Stage	IIA vs. I	0.52	0.19	1.40	0.1953
	IIB vs. I	1.70	0.75	3.87	0.2067
	III vs. I	1.19	0.23	6.15	0.8378
Chemotherapy for rPDAC	Yes vs. No	0.14	0.08	0.24	<.0001
Margin negative resection	Yes vs. No	0.57	0.35	0.93	0.0245
Lymphovascular invasion	Yes vs. No	1.15	0.69	1.92	0.5937
Radiotherapy for rPDAC	Yes vs. No	0.28	0.14	0.53	<.0001
Perioperative therapy	Yes vs. No	1.81	1.02	3.22	0.0417
Ca19–9 level at recurrence	Above 1000 U/ml	1.012	1.002	1.023	0.0237
CA19–9 level at diagnosis	Above 1000 U/ml	1.066	1.014	1.122	0.0131

Variables with *P* value < 0.25 from univariate analysis were included in this model

^a^Perioperative therapy: combination of patients who received neoadjuvant or adjuvant therapy

## Discussion

There is a scarcity of literature on the utilization of chemotherapy among patients with rPDAC after initial curative therapy, and it is not clear the extent to which prior therapy may affect the ability to deliver subsequent chemotherapy. After recurrence of PDAC, only 63% of patients (*N* = 140) in our cohort received chemotherapy, and of those, 13% received only single-agent therapy. The use of more recent, standard combination therapy was associated with longer survival than single-agent or non-standard combination therapy.

An analysis of the SEER database re-iterated the dismal outcomes of mPDAC reporting a mOS of only 2 months. There was an improvement in survival for patients younger than 50 years, and those who were diagnosed in the later period of the study between 2009 and 2013, which may reflect the utilization of multi-agent systemic therapy [16]. Our study, which included a number of patients treated between 2011 and 2014, coincident with the increased utilization of GnP and FOLFIRINOX is in keeping with this hypothesis.

In another study conducted prior to the GnP and FOLFIRINOX era, Meyers and colleagues reported that single agent chemotherapy was administered to 45 of 70 patients with rPDAC (64%) [17]. With a mOS of 10 months, the patients who received therapy seemed to do at least as well as those with de novo mPDAC treated with single agent therapy. A Japanese group also reported outcomes for single agent gemcitabine in recurrent versus de novo metastatic PDAC patients. In their series, recurrent patients (who were never previously exposed to gemcitabine) had longer survival on gemcitabine than did de novo metastatic patients [18]. It is likely that there was a higher prevalence of patients with poor functional status in the de novo metastatic group. Another retrospective review of 40 patients treated in the ‘modern’ era reported a median OS of 16.6 months with chemotherapy for rPDAC. Although numerically superior, it was not statistically different from a median OS of 9.7 months among 141 patients with de novo mPDAC. Of note, only 33% of the rPDAC and 50% of the mPDAC cohort received combination chemotherapy. The treatment regimens employed in this review were FOLFIRINOX, GnP and Gemcitabine and S1 [19].

Our study adds to the literature in several ways. We demonstrate that a significant proportion of patients treated for rPDAC are able to receive multi-agent combination regimens. We also show that the mOS obtained with the approved combinations of FOLFIRINOX or GnP (14 months) is comparable to mOS with these combinations for de novo mPDAC. Additionally, patients seem to derive benefit from chemotherapy at the time of recurrence irrespective of prior perioperative therapy. To date, this is the largest dataset documenting the utilization of modern combination regimens for rPDAC.

Several limitations exist in our study. The retrospective nature of the study meant that potentially important data points were not collected. For example, information on performance status was recorded in only a handful of patients. Given that functional status is an important consideration for offering single agent compared to multiagent therapy, we are unable to exclude selection bias as a significant contributor to the superior outcomes with standard combination chemotherapy agents. Additionally, the reason for not receiving therapy at the time of relapse was not recorded. We therefore were unable to investigate the potential roles of a number of factors including; morbidity from surgical resection and adjuvant therapy, short disease-free interval, rapid progression, comorbidities or patients’ decision on failure to receive therapy. The effect of a favorable/less aggressive disease biology in determining overall survival can therefore not be ruled out. These important questions should be addressed in a prospective manner.

Only 19 patients received single agent chemotherapy in this study and it is difficult to draw strong conclusions from such low numbers. However, double this number received non-standard combination regimens and OS was significantly inferior in this group compared to standard chemotherapy agents. This makes a compelling case for using standard regimens in recurrent PDAC patients considered for combination chemotherapy.

## Conclusion

To our knowledge, this is the largest study describing the outcomes of patients with rPDAC following curative therapy. Our data demonstrate that more than half of patients with rPDAC receive multiagent chemotherapy regimens. The survival outcome reported with modern standard chemotherapy combinations in this study is similar to the outcome with similar regimens in metastatic PDAC. This, therefore, supports the current clinical practice of treating patients with recurrent PDAC similar to those with de novo metastatic disease and provides rationale for inclusion of more of these patients in ‘first line’ clinical trials for metastatic PDAC.

## Additional file

Additional file 1: **Table S1.** Univariate logistic regression of factors associated with not using chemotherapy for rPDAC Variables with a *P* value <0.25 were included in a multivariate analysis. **Table S2.** Multivariate logistic regression of factors associated with not using chemotherapy for rPDAC Factors significant at a *P* value <0.25 on univariate analysis were utilized in the multivariate analysis (DOCX 15 kb)

## Abbreviations

CI: Confidence Interval
EMR: electronic medical record
FOLFIRINOX: 5- fluorouracil/leucovorin/irinotecan/oxaliplatin
FOLFOX: 5-fluorouracil/leucovorin/oxaliplatin
GnP: Gemcitabine and nab-paclitaxel
IRB: institution review board
mOS: median over-all survival
mPDAC: metastatic pancreatic ductal adenocarcinoma
OS: over-all survival
PDAC: Pancreatic ductal; adenocarcinoma
rPDAC: recurrent pancreatic ductal adenocarcinoma
