# Supplementary material for: Overall survival of patients with recurrent pancreatic cancer treated with systemic therapy: a retrospective study
Source: BMC Cancer. 2019 May 17;19:468. doi: 10.1186/s12885-019-5630-4 (PMC6525379; doi:10.1186/s12885-019-5630-4)
Supplement: Supplementary file 1 — Table S1. Univariate logistic regression of factors associated with not using chemotherapy for rPDAC Variables with a P value <0.25 were included in a multivariate analysis. Table S2. Multivariate logistic regression of factors associated with not using chemotherapy for rPDAC Factors significant at a P value <0.25 on univariate analysis were utilized in the multivariate analysis (DOCX 15 kb) [file 12885_2019_5630_MOESM1_ESM.docx]

**Supplementary Data**

**Table 1: Univariate logistic regression of factors associated with not using chemotherapy for rPDAC**

| **Effect** | **Odds Ratio** | **Lower CL** | **Upper CL** | **p-value** |
| --- | --- | --- | --- | --- |
| Gender Female vs Male | 1.63 | 0.92 | 2.89 | 0.0967 |
| Stage IIA v I | 1.43 | 0.24 | 8.38 | 0.9796 |
| Stage IIB v I | 3.06 | 0.65 | 14.41 | 0.9743 |
| Stage III v I | 0.00 | 0.00 | I | 0.9786 |
| Perioperative Chemotherapy Yes vs. no | 0.40 | 0.20 | 0.80 | 0.0092 |
| Margin Negative Yes vs. no | 0.90 | 0.46 | 1.75 | 0.7540 |
| Lymphovascular Invasion Yes vs. no | 1.58 | 0.83 | 2.99 | 0.1633 |
| Perineural Invasion Yes vs. no | 1.14 | 0.49 | 2.65 | 0.7626 |
| Radiotherapy for rPDAC Yes vs. no | 1.56 | 0.65 | 3.77 | 0.3193 |
| Comorbid Conditions Yes vs. no | 1.17 | 0.65 | 2.11 | 0.5968 |
| Diabetes Yes vs No | 1.32 | 0.73 | 2.36 | 0.3555 |
| Site of Recurrence Distant vs. Local | 0.89 | 0.47 | 1.69 | 0.7250 |

| **Effect** | **Odds Ratio** | **Lower CL** | **Upper CL** | ***X*^2^** |
| --- | --- | --- | --- | --- |
| Ca19_9 at recurrence | 1.00 | 1.00 | 1.00 | 0.8969 |
| CA19_9 at diagnosis | 1.00 | 1.00 | 1.00 | 0.1956 |

rPDAC: recurrent pancreatic ductal adenocarcinoma. CL: Confidence Level

**Table 2: *Multivariate logistic regression of factors associated with not using chemotherapy for rPDAC**

| **Effect** | **Odds Ratio** | **Lower CL** | **Upper CL** | **P-value** |
| --- | --- | --- | --- | --- |
| Gender F vs M | 1.95 | 1.02 | 3.70 | 0.0418 |
| Lymphovascular Invasion Yes vs No | 1.84 | 0.91 | 3.71 | 0.0889 |
| Site of recurrence Distant vs Local | 0.84 | 0.41 | 1.70 | 0.6288 |
| Perioperative chemotherapy Yes vs No | 0.47 | 0.21 | 1.05 | 0.0655 |
| CA19_9 at diagnosis | 1.00 | 1.00 | 1.00 | 0.2494 |

rPDAC: recurrent pancreatic ductal adenocarcinoma. CL: Confidence Level

*factors significant at a P value <0.25 on univariate analysis were utilized in the multivariate analysis
